# A single center experience on clinical outcomes of vaginoplasty in cloacal anomaly: a retrospective cohort study

**DOI:** 10.1007/s00383-026-06482-8

**Published:** 2026-08-26

**Authors:** Yuyoung Oh, Honam Hwang, Dayoung Ko, Hee-Beom Yang, Joong Kee Youn, Kwanjin Park, Hoon Kim, Ung Sik Jin, Kwi-Won Park, Hyun-Young Kim

**Affiliations:** 1 Department of Pediatric Surgery, Seoul National University, college of medicine, 28 Yeongeon-dong, Jongno-gu, Seoul, 110-744 South Korea; 2 Department of Surgery, Seoul National University College of Medicine, Seoul, Korea; 3 Department of Surgery, Seoul National University Bundang Hospital, Seongnam, Korea; 4 Department of Urology, Seoul National University Children’s Hospital, Seoul, Korea; 5 Department of Obstetrics and Gynecology, Seoul National University Hospital, Seoul, Korea; 6 Department of Plastic and Reconstructive surgery, Seoul National University Hospital, Seoul, Korea; 7 Department of Surgery, Chung-Ang University College of Medicine, Seoul, Korea

**Keywords:** Cloaca, Vagina, Anorectal malformations, Anus, Imperforate

## Abstract

**Purpose:**

This study analyzed the demographics and clinical outcomes of vaginoplasty in patients with cloacal anomaly, focusing on the impact of surgical timing, method, and common channel length. Subgroup analyses evaluated the influence of timing across anatomical categories.

**Methods:**

We retrospectively reviewed 28 patients who underwent vaginoplasty at Seoul National University Children’s Hospital between 1989 and 2024. Data included associated anomalies, common channel length, timing and method of vaginoplasty, and outcomes related to menstruation, sexual activity, vaginal complications, bowel function, and urinary function. Patients were classified into three groups according to timing of vaginoplasty: Group A (simultaneous with anorectoplasty, *n* = 21), Group B (separate surgery before age 11, *n* = 3), and Group C (separate surgery after age 11, *n* = 4). Group A included both short and long common channel patients. Subgroup analysis was performed based on common channel length (≥ 3 cm vs. <3 cm).

**Results:**

The median age at vaginoplasty was 12.5 months and pull-through was performed in 26 patients. The median age at anorectoplasty was 9 months in Group B and 13 months in Group C. Vaginal separation was performed at anorectoplasty in two of three Group B patients and one of four Group C patients, while the remaining patients underwent delayed vaginal separation. Menstruation occurred in 80% of post-pubertal patients, and 9.1% reported sexual intercourse. Group C showed higher rates of vaginal complications, longer operative times, and increased urinary tract infections. In subgroup analysis restricted to patients with long common channels, similar trends were observed.

**Conclusion:**

While functional outcomes were generally favorable, among patients with long common channels, delayed vaginoplasty after puberty was associated with higher rates of vaginal complications. These findings suggest that surgical timing may influence outcomes in this subgroup, although the retrospective design limits causal inference.

## Introduction

Cloacal anomaly is a rare and complex congenital malformation where the urethra, vagina, and rectum converge into a single perineal opening [1]. Classified as a high-type anorectal malformation (ARM) under the Wingspread and Peña classifications, it represents about 10% of female ARM cases [2–4]. The main objectives of surgical management are to achieve urinary and fecal continence, preserve sexual function, and ensure future fertility potential. Vaginoplasty plays a crucial role in meeting these goals, with common surgical techniques including vaginal pull-through, switch maneuver, bowel segment replacement, and local flap reconstruction [5, 6].

Treatment strategies typically begin with a protective colostomy, followed by either a one-stage anorectourethroplasty combined with vaginoplasty or a staged approach that delays vaginoplasty. While the timing of anorectoplasty is generally well established, the optimal timing for vaginoplasty is still debated. A limited number of retrospective studies have explored this issue, although timing has not always been analyzed as a primary factor and the methodologies remain heterogeneous [6, 7]. A major limitation is the methodological heterogeneity, particularly the vague or overly broad definitions of “early” and “late” vaginoplasty, which complicates meaningful comparisons.

The controversy surrounding cloacal anomalies is further complicated by significant anatomical variability, particularly in the length of the common channel and related urogenital structures. These variations hinder standardized comparisons across patients, leading to decisions about timing that often rely on the surgeon’s clinical judgment rather than established protocols. While several large-scale international studies have examined outcomes in cohorts of up to 570 patients with cloacal anomalies [5, 7], data from Korea remain limited. Furthermore, few studies, both globally and domestically, have specifically investigated the relationship between the timing of vaginoplasty and postoperative functional outcomes.

This study aims to fill these gaps by analyzing the demographic and clinical outcomes of vaginoplasty in patients with cloacal anomalies. We will compare surgical outcomes based on the timing of vaginoplasty, the method used, and the length of the common channel, with subgroup analyses categorized by common channel length. Acknowledging the heterogeneity of anatomical features and the multifactorial influences involved, we seek to derive meaningful insights into how timing may impact outcomes, ultimately aiming to inform future clinical decision-making and enhance long-term results.

## Methods

**Fig. 1:**
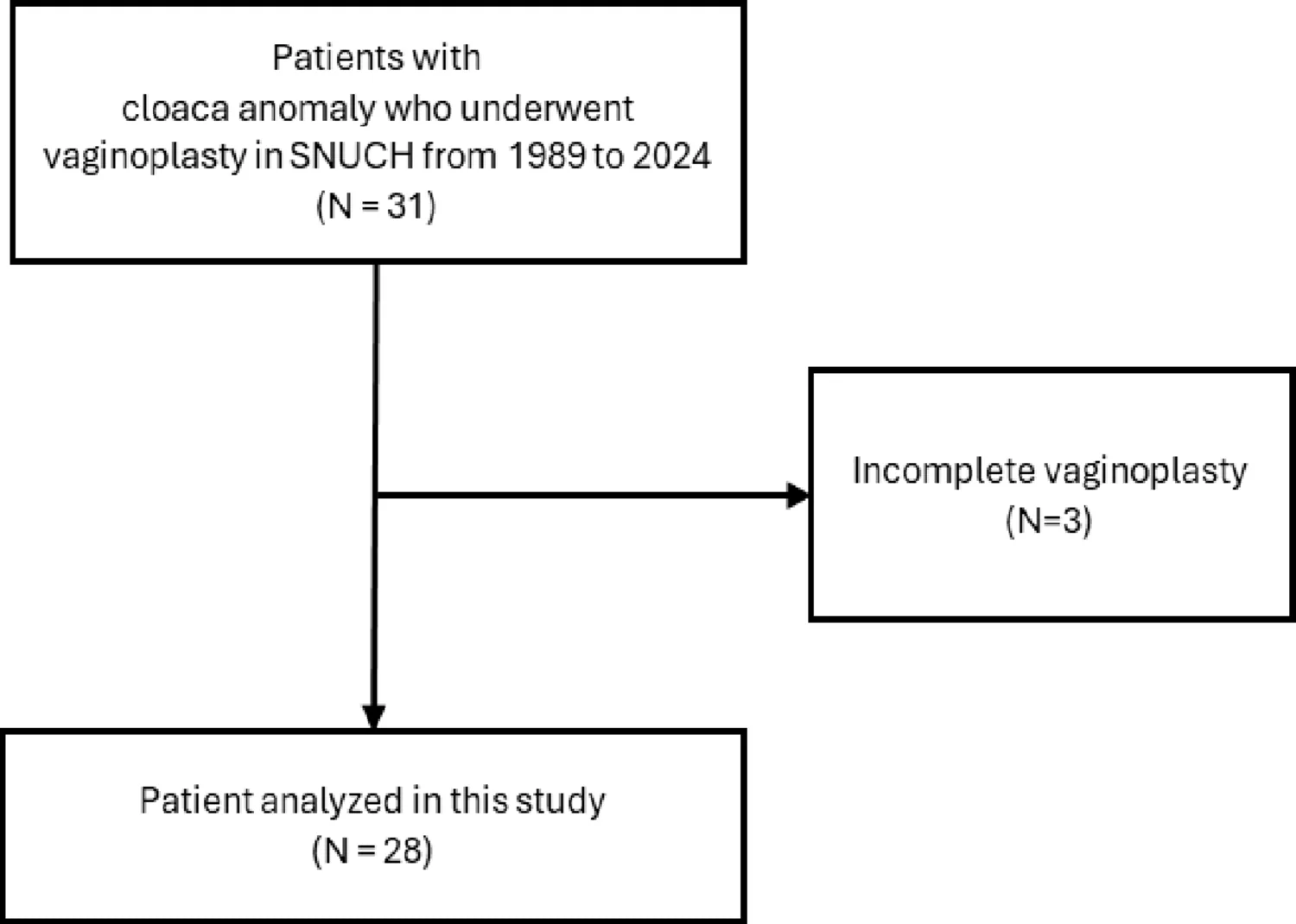
Flow diagram of patient selection. A total of 31 patients with cloacal anomalies who underwent vaginoplasty at Seoul National University Children’s Hospital between 1989 and 2024 were initially identified. Three patients were excluded due to incomplete vaginoplasty, defined as cases with insufficient urethral length resulting in a persistent common channel. The final study cohort consisted of 28 patients included in the analysis

We conducted a retrospective study of patients with cloacal anomalies who underwent vaginoplasty at Seoul National University Children’s Hospital between 1989 and 2024. Out of 31 patients, three cases were classified as failed vaginoplasty, defined as procedures where the urethral length was insufficient, resulting in a persistent common channel. Excluding these cases, a total of 28 patients were included in the final study cohort. The patient selection process is summarized in Fig. 1. The Institutional Review Board of Seoul National University Hospital (IRB No. 1604-081-754) approved all data collection and analysis, and informed consent was waived due to the retrospective nature of the study.

The mean follow-up period was 21 years, with the longest follow-up reaching 35 years. Due to the retrospective nature of the study and the long study period, loss to follow-up could not be reliably quantified. For each patient, we collected data on current age, gestational age, birth weight, associated anomalies, common channel length, timing and technique of vaginoplasty, operative time, and need for reoperation. Functional outcomes assessed included menstruation, sexual intercourse capabilities, vaginal complications, bowel function, and urinary function. Additionally, when available, we recorded information on marriage and childbirth.

Initially, patients were analyzed based on the length of the common channel, with 13 classified as having a long channel (≥ 3 cm) and 15 having a short channel (< 3 cm), using the commonly accepted threshold reported in previous studies [8]. Common channel length was primarily assessed using preoperative contrast genitography (cloacagram). The measurement was defined as the distance from the perineal opening to the point of divergence between the urethra and vagina. When available, findings from cystoscopy and vaginoscopy were used as adjuncts to better define the anatomical configuration. Given the retrospective nature of the study and the long study period, measurements were not obtained using a standardized protocol in all cases and were therefore considered approximate anatomical estimates rather than precise values.

**Fig. 2:**
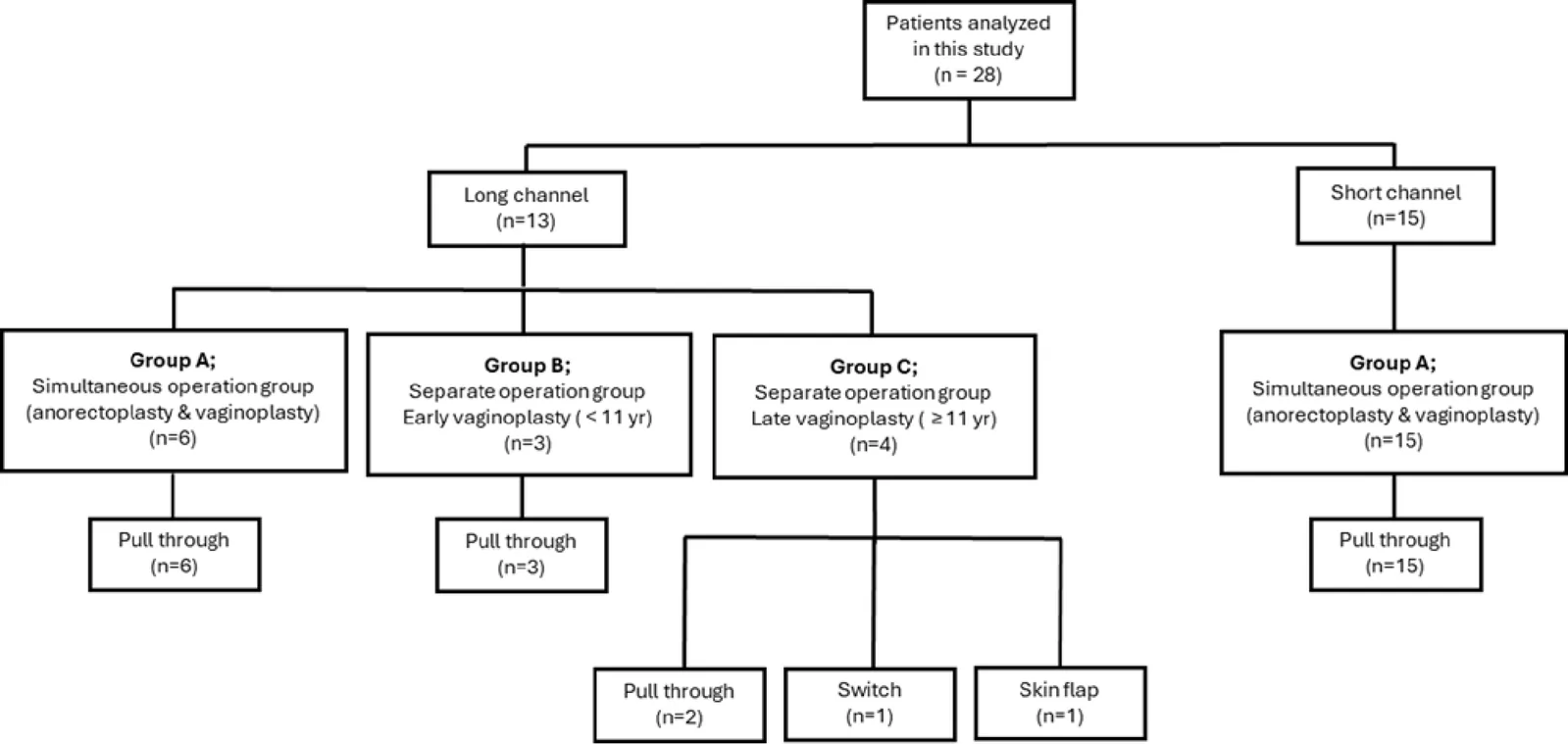
Flowchart showing the classification of 28 patients with a cloacal anomaly based on the timing of vaginoplasty and common channel length. Patients were stratified by common channel length: long channel (≥ 3 cm) (*n* = 13) and short channel (< 3 cm) (*n* = 15). Each group was further divided into three categories based on timing: Group A (*n* = 21), where vaginoplasty was performed simultaneously with anorectoplasty; Group B (*n* = 3), where early separate vaginoplasty was performed before age 11; and Group C (*n* = 4), where late separate vaginoplasty was performed after age 11

Patients were then grouped according to the timing of vaginoplasty: Group A included those who underwent simultaneous anorectourethroplasty and vaginoplasty (*n* = 21); All patients in Group A underwent posterior sagittal anorectovaginourethroplasty (PSARVUP), and no patients underwent total urogenital mobilization (TUM) in the final study cohort. Cases in which complete vaginal reconstruction was not achieved were classified as incomplete vaginoplasty and were excluded from the analysis (*n* = 3). Group B comprised patients who had staged vaginoplasty before puberty (≤ 11 years of age, *n* = 3); and Group C included those who had staged vaginoplasty after puberty (> 11 years of age, *n* = 4), with 11 years set as the cutoff age to reflect the typical onset of puberty in females. Each group was further subdivided based on the length of the common channel, categorized as long (≥ 3 cm) and short (< 3 cm) (Fig. 2).

We began by analyzing the overall demographic and clinical characteristics of the participants. Next, we compared these characteristics across three groups based on vaginoplasty timing, method, and common channel length. Functional outcomes were evaluated for the entire cohort and compared between groups. Additionally, we conducted subgroup analyses within the long-channel cohorts to assess whether the timing of vaginoplasty influenced clinical outcomes. Although surgeries were performed by different surgeons, the distribution among groups was relatively even, and this variable was not included in the statistical analysis.

Statistical analyses were conducted using SPSS version 7.1 for Windows (IBM Corp., Armonk, NY, USA). Fisher’s exact test was employed to compare categorical variables, and the corresponding p-values were calculated when applicable. For comparisons involving more than two groups, one-way analysis of variance (ANOVA) was utilized. Normality was assessed using the Shapiro–Wilk test and homogeneity of variances using Levene’s test. When assumptions were violated, the Kruskal–Wallis test was used as a non-parametric alternative. A p-value of less than 0.05 was considered statistically significant.

## Results

**Fig. 3:**
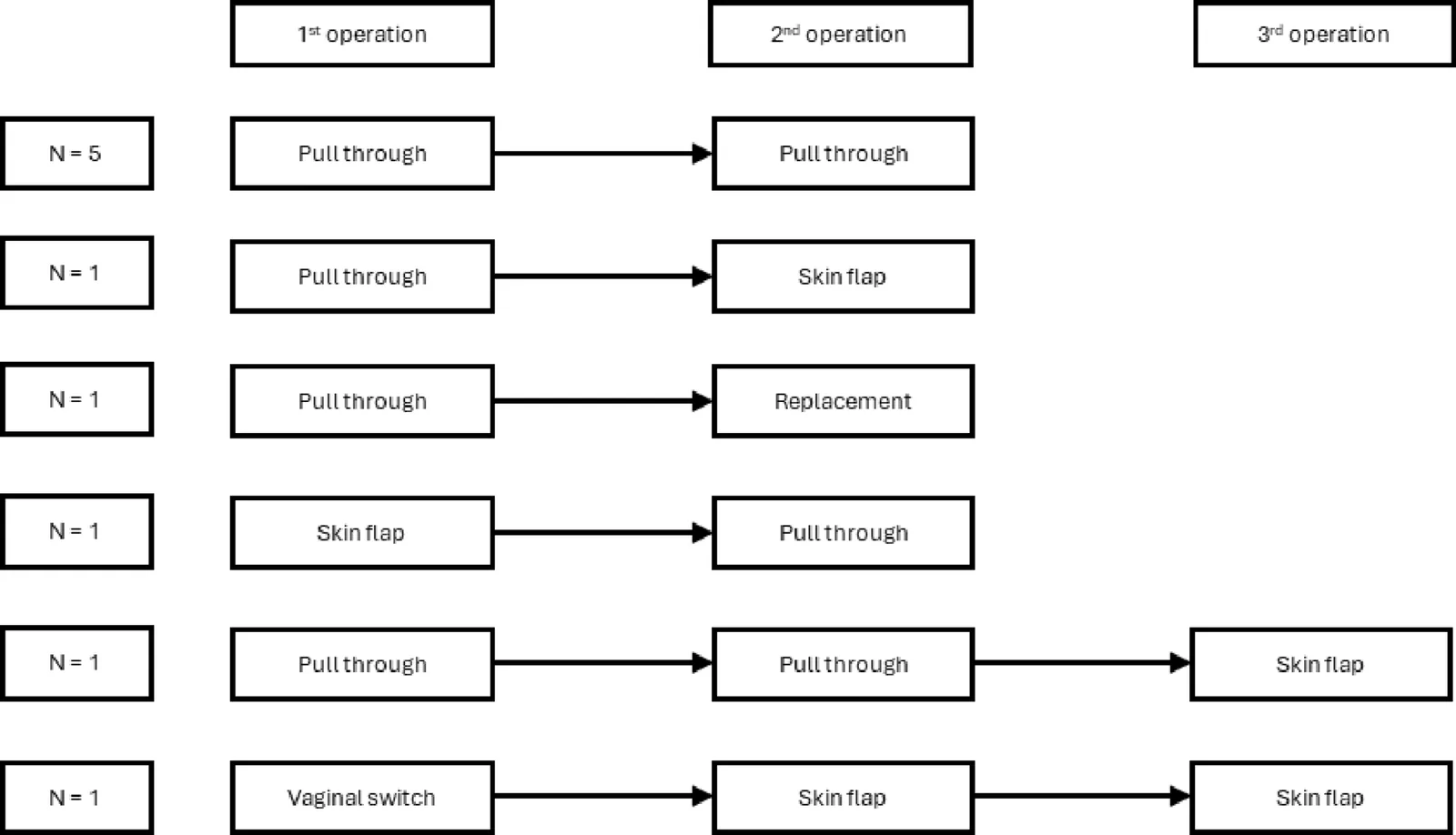
Flow chart of re-vaginoplasty procedures in patients with cloacal anomalies. A total of 10 patients underwent re-vaginoplasty, with up to three revision surgeries performed. The chart illustrates the surgical methods used at each stage of reoperation, including pull-through, switch maneuver, bowel segment replacement, and local flap reconstruction. Numbers in parentheses indicate the number of patients who underwent each technique

Table 1 summarizes the demographic statistics of the 28 patients, categorizing them based on common channel length (long vs. short). The median current age was 21 years, with the oldest patient being 35. Among the congenital anomalies, Müllerian anomalies were the most prevalent, affecting 25 patients (86.2%). The overall mean common channel length was approximately 3 cm, with the long channel group averaging 5 ± 2 cm and the short channel group averaging 2 ± 1 cm (*p* < 0.001). The median age at first vaginoplasty was 12.5 months (range, 3–279), with an average of 29 months (range, 3–279) in the long channel group and 12 months (range, 7–23) in the short channel group (*p* = 0.012). Ten patients underwent reoperation, with some having up to three procedures. The surgical techniques for the first, second, and third vaginoplasty are detailed in the table, with pull-through being the most commonly used method (26 cases). A flow chart (Fig. 3) illustrates the surgical techniques employed for each redo procedure among the 10 patients who required re-vaginoplasty.

**Table 1:** Demographics according to common channel length

	*N* (%)			
	Total (*n* = 28)	Long channel (*n* = 13)	Short channel (*n* = 15)	*P* value
Current age (year, median, range)	21 (1–35)	20 (4–35)	21 (1–34)	0.223
Gestational age (week, mean ± SD)	37.4 ± 3.2	36.6 ± 3.9	38.0 ± 2.4	0.250
Preterm	10 (35.7)	6 (46.2)	4 (26.7)	0.433
Birth weight (kg, mean ± SD)	2.7 ± 0.6	2.7 ± 0.7	2.7 ± 0.5	0.861
Low birth weight	6 (21.4)	2 (15.4)	4 (26.7)	1.000
Congenital anomaly				
Cardiac	11 (37.9)	4 (37.8)	7 (46.7)	0.460
Gastrointestinal	4 (13.8)	2 (15.4)	2 (13.3)	1.000
Spinal/Vertebral	10 (34.5)	4 (37.8)	6 (40.0)	0.705
Renal	5 (17.2)	3 (23.1)	2 (13.3)	0.639
Mullerian^a^	25 (86.2)	12 (92.3)	13 (86.7)	1.000
Others^b^	3 (10.3)	2 (15.4)	1 (6.7)	0.583
Common channel (cm, mean ± SD)	3 ± 2	5 ± 2	2 ± 1	< 0.001^*^
Age of anorectoplasty (month, median, range)	10 (1-28)	9 (1-28)	12 (7-23)	0.196
Age of 1st vaginoplasty (month, median, range)	12.5 (3-279)	29 (3-279)	12 (7–23)	0.012^*^
Operation time of 1st vaginoplasty (min., mean ± SD)	388.0 ± 257.4	554.7 ± 247.2	204.7 ± 89.8	< 0.001^*^
Method of 1st vaginoplasty (*n* = 28)				0.206
Pull-through	26	11	15	
Switch	1	1	0	
Replacement	0	0	0	
Flap	1	1	0	
Method of 2nd vaginoplasty (*n* = 10)				1.000
Pull-through	7	4	3	
Switch	0	0	0	
Replacement	1	0	1	
Flap	2	1	1	
Method of 3rd vaginoplasty (*n* = 2)				0.206
Pull-through	0	0	0	
Switch	0	0	0	
Replacement	0	0	0	
Flap	2	2	2	
Re-vaginoplasty	10	5	5	1.000

^*^P < 0.05, statistically significant

^a^Müllerian anomalies include uterine didelphys (*n* = 18), longitudinal vaginal septum (*n* = 1), unicornuate uterus (*n* = 1), complex anomalies (*n* = 4; unicornuate uterus with double vagina (*n* = 1), uterine didelphys with unilateral vaginal atresia (*n* = 2), vaginal duplication with hemiuterus (*n* = 1)), NFC(not further classifiable) (*n* = 1; This patient underwent a simultaneous operation (PSARVUP) and was noted to have double vagina. However, the uterine structure was not documented, and the patient was lost to follow-up after 2017)

^b^Other anomalies include vascular malformation of the cheek, congenital hypoplasia of the thumb, and hypoplastic bilateral acoustic canals

**Fig. 4:**
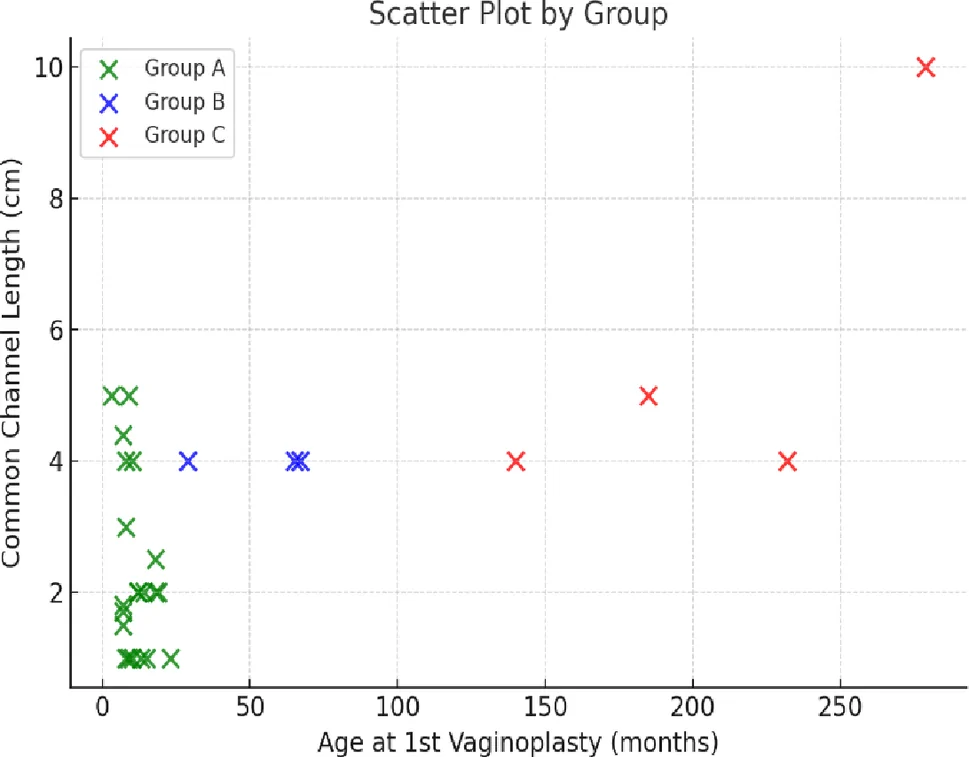
Scatter plot illustrating the relationship between age at first vaginoplasty (in months) and common channel length (in centimeters) for each patient. Patients are categorized into surgical timing groups: Group A (green, simultaneous vaginoplasty), Group B (blue, early separate vaginoplasty before age 11), and Group C (red, late vaginoplasty after age 11). The horizontal blue line indicates the 3 cm threshold used to differentiate between long and short common channels. The vertical green line marks the age cutoff at 11 years (132 months), which is used to distinguish between early and late vaginoplasty

A comparison of age at first vaginoplasty and common channel length across Groups A, B, and C is presented in Table 2 and illustrated in Fig. 4. The mean common channel lengths were 2 ± 1 cm for Group A, 4 ± 0 cm for Group B, and 6 ± 3 cm for Group C, with Group C exhibiting significantly longer channels (*p* = 0.001). The median age at first vaginoplasty was 10 months for Group A, 65 months for Group B, and 208.5 months for Group C (*p* < 0.001). Given that group classification was based on age, these results were anticipated, indicating that the stratification was appropriately conducted. Mean operation times also differed significantly: 250.6 min for Group A, 502 min for Group B, and 783.5 min for Group C (*p* < 0.001). All patients in Groups A and B underwent pull-through vaginoplasty, while patients in Group C also received switch or flap techniques.

**Table 2:** Demographics according to the timing of vaginoplasty

	*N* (%)			
	Group A (*n* = 21)	Group B (*n* = 3)	Group C (*n* = 4)	*p* value
Current age (year, median, range)	21 (1–34)	22 (19–26)	29.5 (20–35)	0.176
Gestational age (week, mean ± SD)	37.2 ± 3.0	35 ± 5.6	40 ± 0	0.115
Preterm (N (%))	8 (38.1)	2 (66.6)	0 (0)	0.433
Birth weight (kg, mean ± SD)	2.7 ± 0.5	2.4 ± 1.2	3.1 ± 0.6	0.367
Low birth weight (N (%))	5 (23.8)	1 (33.3)	0 (0)	1.000
Congenital anomaly				
Cardiac	10 (47.6)	1 (33.3)	0 (0)	0.460
Gastrointestinal	3 (14.3)	1 (33.3)	0 (0)	1.000
Spinal/Vertebral	8 (38.1)	1 (33.3)	1 (25.0)	0.705
Renal	3 (14.3)	1 (33.3)	1 (25.0)	0.639
Mullerian	18 (85.7)	3 (100)	4 (100)	1.000
Others	3 (14.3)	0 (0)	0 (0)	0.583
Common channel (cm, mean ± SD)	2 ± 1	4 ± 0	6 ± 3	0.001^*^
Age of anorectoplasty (month, median, range)	10 (3–23)	9 (5–23)	13 (1–28)	0.853
Age of 1st vaginoplasty (month, median, range)	10 (3–23)	65 (29–67)	208.5 (140–279)	< 0.001^*^
Operation time of 1st vaginoplasty (min., mean ± SD)	250.6 ± 112	502 ± 284.2	783.5 ± 154.8	< 0.001^*^
Method of 1st vaginoplasty				0.206
Pull-through	21	3	2	
Switch	0	0	1	
Replacement	0	0	0	
Flap	0	0	1	
Re-vaginoplasty	6 (28.6)	1 (33.3)	3 (75)	1.000

^*^P < 0.05, statistically significant

All patients in Groups B and C underwent anorectoplasty earlier in life. The median age at anorectoplasty was 9 months (range, 5–23) in Group B and 13 months (range, 1–28) in Group C. In Group B, vaginal separation was performed at the time of anorectoplasty in two patients, while in one patient the confluence remained intact until later vaginoplasty; in Group C, vaginal separation was performed in one patient, with the remaining three undergoing delayed separation.

Table 3 shows that seven vaginal complications were observed in total. Of these, five cases involved vaginal stenosis or stricture, which required mold insertion or vaginal dilation, while two cases involved recurrent urethrovaginal fistulas due to ruptures in the anterior vaginal wall, necessitating reoperation. Among the 28 patients, 25 were older than 11 at the time of the last follow-up, and of these, 20 had experienced menstruation. Among the five who had not, four were lost to follow-up after the premenarcheal period, and one was diagnosed with primary amenorrhea and received GnRH agonist therapy. Two patients reported engaging in sexual intercourse: one had normal sexual function, while the other experienced a perineal tear. One patient was married, but none became pregnant during the follow-up period.

In terms of bowel function, all but three patients were able to have voluntary bowel movements. Among these three, two were under the age of 5 at the last follow-up, and one required a permanent end ileostomy due to serosal tearing during redo pull-through surgery. For urinary function, 24 patients (82.8%) were able to self-void, while four required catheterization, including those with a Mitrofanoff channel or those needing intermittent catheterization (CIC).

Regarding functional outcomes based on common channel length, the long channel group exhibited a higher rate of urinary complications necessitating catheterization compared to the short channel group (*p* = 0.035) (Table 3). When analyzed by surgical timing groups (A, B, C), as shown in Table 4, vaginal complications were observed in 14.3% of Group A, 33.3% of Group B, and 75.0% of Group C (*p* = 0.018). Experiences related to sexual intercourse were reported as 0%, 0%, and 50% for each group, respectively (*p* = 0.025). While there were no significant differences in bowel function across groups, urinary complications requiring catheterization were reported at rates of 0%, 66.7%, and 50%, respectively (*p* = 0.001). Notably, three cases of urinary tract infection (including cystitis and pyelonephritis) occurred solely in Group C (75%), all of which occurred in the postoperative period following vaginoplasty.

**Table 3:** Functional outcomes of vaginoplasty according to common channel length

		*N* (%)			
		Total (*n* = 28)	Long channel (*n* = 13)	Short channel (*n* = 15)	*P* value
Age of anorectoplasty (month, median, range)		10 (1–28)	9 (1–28)	12 (7–23)	0.196
Age of 1st vaginoplasty (month, median, range)		12.5 (3–279)	29 (3–279)	12 (7–23)	0.012^*^
Vaginal complications^a^					0.519
Vaginal stenosis/stricture		5 (17.9)	3 (23.1)	2 (13.3)	0.639
Recurrence of U-V fistula		2 (7.1)	1 (7.7)	1 (6.7)	1.000
Menstruation					
≤ 11 year (*n* = 3)		0/3 (0)	0/2 (0)	0/1 (0)	–
> 11 year (*n* = 25)		20^b^/25 (80)	9/11 (81.8)	11/14 (78.6)	0.661
Sexual intercourse					
≤ 17 year (*n* = 6)		0/6 (0)	0/5 (0)	0/1 (0)	–
> 17 year (*n* = 22)		2^c^/22(9.1)	2/9 (22.2)	0/14 (0)	0.121
Marriage		1 (3.5)	1 (7.7)	0 (0)	0.464
Childbearing		0 (0)	0 (0)	0 (0)	–
Bowel function					
Voluntary bowel movement					0.583
Yes		25 (89.3)	11 (84.6)	14 (93.3)	
No^d^		3 (10.7)	2 (15.4)	1 (6.7)	
Urinary function					0.035^*^
Self-voiding		24 (82.8)	9 (69.2)	15 (100)	
Urinary catheterization^e^		4 (14.3)	4 (30.8)	0 (0)	
Urinary tract infection (APN, cystitis)		3 (10.7)	3 (23.1)	0 (0)	0.087

^*^P < 0.05, statistically significant

^a^U–V fistula recurrence refers to cases where a fistula reformed due to rupture of the anterior vaginal wall

^b^Among the five patients counted as having no menstruation (although they are older than 11), four were lost to follow-up after the premenarcheal period, and one had primary amenorrhea and was under treatment with a GnRH agonist

^c^Among the two patients who had experienced sexual intercourse, one reported normal sensation, while the other one also had normal sensation but required perineal suturing due to a tear sustained during intercourse. Among the 20 patients without sexual experience, one was advised not to engage in sexual intercourse due to physical impossibility

^d^Among the three patients for whom voluntary bowel movement was not possible, two patients were under the age of 5, one had a permanent end ileostomy due to serosal tearing occurred during attempted redo-pull-through surgery

^e^Four patients: Mitrofanoff state (*n* = 3); clean intermittent catheterization (CIC), intermittent or continuous, required (*n* = 1)

**Table 4:** Functional outcome of vaginoplasty according to the timing of operation

	*N* (%)			
	Group A (*n* = 21)	Group B (*n* = 3)	Group C (*n* = 4)	*p* value
Vaginal complications				0.018^*^
Vaginal stenosis/stricture	2 (9.5)	0 (0)	3 (75.0)	0.015^*^
Recurrence of U-V fistula	1 (4.8)	1 (33.3)	0 (0)	1.000
Menstruation				
≤ 11 year (*n* = 3)	0/3 (0)	0/0 (0)	0/0 (0)	–
> 11 year (*n* = 25)	14/18 (77.8)	3/3 (100)	3/4 (75)	0.788
Sexual intercourse				
≤ 17 year (*n* = 6)	0/6 (0)	0/0 (0)	0/0 (0)	–
> 17 year (*n* = 22)	0/15 (0)	0/3 (0)	2/4 (50)	0.026^*^
Marriage	0	0	1 (25)	0.143
Childbearing	0	0	0	–
Bowel function				
Voluntary bowel movement				1.000
Yes	19 (90.5)	2 (66.7)	4 (100)	
No	2 (9.5)	1 (33.3)	0 (0)	
Urinary function				0.001^*^
Self-voiding	21 (100)	1 (33.3)	2 (50)	
Urinary catheterization	0 (0)	2 (66.7)	2 (50)	
Urinary tract infection (APN, cystitis)	0 (0)	0 (0)	3 (75)	0.001^*^

^*^P < 0.05, statistically significant

When functional outcomes were analyzed by the method of vaginoplasty, no significant differences were found in other outcomes. However, the incidence of urinary tract infection (UTI) was lowest in the pull-through group at 3.8%, while all patients who underwent vaginoplasty using the switch or flap methods (one patient each) experienced a UTI (Table 5).

**Table 5:** Functional outcome according to the method of 1st vaginoplasty

	*N* (%)			
	Pull through (*n* = 26)	Switch (*n* = 1)	Flap (*n* = 1)	*p* value
Vaginal complications				0.250
Vaginal stenosis/stricture	4 (15.4)	0 (0)	1 (100)	0.179
Recurrence of U-V fistula	2 (7.7)	0 (0)	0 (0)	1.000
Menstruation				
≤ 11 year (*n* = 3)	0/3 (0)	0/0 (0)	0/0 (0)	–
> 11 year (*n* = 25)	18/23 (78.3)	1/1 (100)	1/1 (100)	0.810
Sexual intercourse				
≤ 17 year (*n* = 6)	0/6 (0)	0/0 (0)	0/0 (0)	–
> 17 year (*n* = 22)	0/20 (0)	1/1 (100)	1/1 (100)	0.004^*^
Marriage	0	1	0	0.140
Childbearing	0	0	0	–
Bowel function				
Voluntary bowel movement				1.000
Yes	23 (88.5)	1 (100)	1 (100)	
No	3 (11.5)	0 (0)	0 (0)	
Urinary function				1.000
Self-voiding	23 (88.5)	0 (0)	1 (100)	
Urinary catheterization	3 (11.5)	1 (100)	0 (0)	
Urinary tract infection (APN, cystitis)	1 (3.8)	1 (100)	1 (100)	0.008^*^

^*^P < 0.05, statistically significant

To minimize the influence of anatomical differences, a subgroup analysis was conducted for the long channel groups. As indicated in Table 6, among the 13 patients in the long channel group, Group C again demonstrated the highest rates of vaginal complications (0%, 33.3%, 75%, *p* = 0.018) and UTI (0%, 0%, 75%, *p* = 0.014).

**Table 6:** Subgroup analysis of functional outcomes by timing of vaginoplasty among patients with a long common channel (Total = 13)

	Group A in long channel patients	Group B in long channel patients	Group C in long channel patients	*p* value
Number of patients	6	3	4	–
Common channel (cm, mean ± SD)	4 ± 1	4 ± 0	6 ± 3	0.316
Age at anorectoplasty (month, median, range)	8 (3–10)	9 (5–23)	13 (1–28)	0.436
Age at vaginoplasty (month, median, range)	8 (3–10)	65 (29–67)	208.5 (185–279)	< 0.001^*^
Method of 1st vaginoplasty				0.077^*^
Pull-through	6	3	1	
Switch	0	0	1	
Replacement	0	0	0	
Flap	0	0	1	
Vaginal complications				0.018^*^
Vaginal stenosis/stricture	0 (0.0)	0 (0)	3 (75)	0.014^*^
Recurrence of U-V fistula	0 (0.0)	1 (33.3)	0 (0)	1.000
Menstruation				
≤ 11 year (*n* = 2)	0/2 (0)	0/0 (0)	0/0 (0)	–
> 11 year (*n* = 11)	3/4 (75)	3/3 (100)	3/4 (75)	1.000
Sexual intercourse				
≤ 17 year (*n* = 5)	0/5 (0)	0/0 (0)	0/0 (0)	–
> 17 year (*n* = 8)	0/1 (0)	0/3 (0)	2/4 (50)	0.321
Marriage	0	0	1	–
Childbearing	0	0	0	–
Bowel function				
Voluntary bowel movement				0.654
Yes	5 (83.3)	3 (75)	4 (100)	
No	1 (16.7)	1 (25)	0 (0)	
Urinary function				0.098
Self-voiding	6/6 (100)	1/3 (33.3)	2/4 (50)	
Urinary catheterization	0/6 (0)	2/3 (66.7)	2/4 (50)	
Urinary tract infection (APN, cystitis)	0/6 (0)	0/3 (0)	3/4 (75)	0.014^*^

^*^P < 0.05, statistically significant

## Discussion

This study aimed to analyze how the timing of vaginoplasty affects functional outcomes in patients with cloacal anomalies, particularly concerning common channel length. Although several previous studies have addressed this topic, most involved heterogeneous patient populations and used varying definitions of “early” and “late” vaginoplasty, which limits their relevance to cloacal anomalies.

In our cohort, patients who underwent vaginoplasty after puberty (Group C) experienced the highest rates of vaginal complications and urinary tract infections. Additionally, Group C had the longest average common channel length, the most extended operating time, and the greatest use of surgical techniques beyond the standard pull-through procedure. These findings indicate that patients with more anatomically complex malformations may face delays in surgery, which could affect both the choice of surgical technique and postoperative outcomes.

Overall, functional outcomes in the cohort were generally favorable. Menstruation was reported in 80% of post-pubertal patients, surpassing the 32–66% range noted in previous studies [2, 9, 10]. This percentage might be even higher if we exclude those lost to follow-up before puberty. Among patients without menstruation despite being older than 11 years, four were lost to follow-up after the premenarcheal period and one had primary amenorrhea. The incidence of vaginal stenosis or stricture was 25%, aligning with the 22–36% range reported for obstructed menstruation, where introital stenosis is the most common cause. Primary amenorrhea was rare, consistent with prior literature that indicates preserved ovarian function in most cloacal patients [2, 9]. Sexual activity was reported by 9.1% of patients of appropriate age, which is lower than the 57–65% reported in Western studies [10]; this discrepancy may reflect differences in sociocultural context or patient reluctance to discuss sexual history. Only one patient was married, and no pregnancies were reported, although the median follow-up age was 21 years, suggesting that longer-term data are necessary. Müllerian anomalies are common in cloacal anomalies, and pregnancies in these patients often require surgical intervention; complications such as preterm labor, miscarriage, and urinary tract infections have been documented [2].

Bowel function outcomes were favorable, with 89.3% of patients achieving voluntary control. This rate is significantly higher than the 43–56% control rates reported in some studies [11–13]. However, the literature shows a wide range of fecal incontinence and constipation rates (11–89% and 0–93%, respectively) [2, 14–18], which limits the prognostic value. Our study found no significant differences in bowel outcomes based on the timing of vaginoplasty or channel length, so a detailed subgroup analysis was not within our scope. Urinary function outcomes also compared favorably with previous reports, showing high rates of social continence and a lower need for clean intermittent catheterization (CIC) than the 49–67% reported in earlier studies [14, 19]. Previous research has indicated conflicting associations between common channel length and urinary continence [2, 19].

In summary, the functional outcomes in our study were generally comparable to or slightly better than those reported in existing literature. However, these outcomes may vary based on the patient’s anatomical characteristics and the complexity of the malformation.

A key strength of this study is our effort to isolate the effect of vaginoplasty timing by stratifying patients according to common channel length. By dividing patients into short and long channel groups and analyzing outcomes based on the timing of surgery within each group, we aimed to minimize confounding factors related to anatomical complexity. Importantly, as Group A included both short- and long-channel patients, direct comparisons across Groups A, B, and C may be confounded by anatomical heterogeneity. Therefore, the primary focus of our analysis was on subgroup comparisons within patients with long common channels. Notably, within the long channel group, patients in Group C—those who underwent vaginoplasty after puberty—exhibited the highest complication rates, emphasizing that this finding is not solely explained by anatomical severity and suggesting a potential independent effect of surgical timing.

Group C included an exceptional case with a 10 cm common channel, which likely contributed to the higher average channel length observed in this group, despite the small sample size (*n* = 4). Since the current cut-off for short and long channels is set at 3 cm, this outlier raises the possibility that a more refined classification—one that further differentiates long channels beyond 3 cm—could enhance outcome prediction. Future studies may consider proposing a revised threshold to better capture the spectrum of anatomical severity.

Additionally, the initial vaginoplasty techniques varied in Group C, where procedures like the switch maneuver or flap reconstruction were utilized instead of the standard pull-through approach. This variation is likely due to the significantly longer channel lengths and associated poor vascularity in Group C, which necessitated alternative surgical strategies. For example, the patient with the 10 cm common channel underwent a switch maneuver, likely the only viable option. Although this patient experienced a urinary tract infection, they did not develop vaginal stenosis or urethrovaginal fistula, probably due to the use of a non-pull-through technique. These findings suggest that the higher rate of vaginal complications in Group C cannot be solely attributed to channel length. In fact, this extraordinarily long-channel case did not present any vaginal complications, challenging the assumption that longer channels inherently predict poorer vaginal outcomes.

In patients with a long common channel or those who underwent vaginoplasty at a later age, there was a higher incidence of urinary complications, including urinary tract infections and the need for urinary catheterization. In contrast, no significant differences in vaginal complications were found between the long and short channel groups. However, subgroup analysis within the long channel group —where anatomical heterogeneity is minimized— revealed that Group C experienced a significantly higher rate of vaginal complications. These findings suggest that, within anatomically comparable patients, the timing of vaginoplasty may have a greater impact on vaginal complications than the length of the common channel itself. In addition, urinary outcomes may be influenced by the choice of surgical technique and the management of the common channel, including whether it is incorporated into vaginal reconstruction or preserved for urethral function. However, as all patients in this cohort underwent PSARVUP and no TUM cases were included, this aspect could not be directly evaluated and warrants further investigation in future studies.

Nevertheless, caution is warranted in interpreting these results. In any retrospective study, it is challenging to completely rule out the possibility that the timing of surgery was influenced by the inherent anatomical complexity of each case. Consequently, the higher complication rates observed in patients who underwent vaginoplasty later (Group C) may be linked not only to the delay but also to the more complex anatomical conditions that necessitated the later surgery. This presents a classic “chicken-and-egg” dilemma. While the study aimed to isolate the effect of timing, the conclusions drawn are limited. Additionally, parental preferences—such as a desire for early correction or delaying surgery until the child was older—may have influenced surgical timing. Although operative times were significantly longer in Group C, it remains unclear whether the extended duration of surgery contributed to complications or if the more complex anatomy resulted in longer surgery times and an increased risk of complications. This study cannot definitively answer this question.

In addition, the interpretation of common channel length as a surrogate marker of initial anatomical severity should be approached with caution. Because the common channel may increase in length with age and pelvic growth, measurements obtained at later ages may reflect developmental changes rather than the original anatomical complexity present at birth. Given the age differences among the study groups, longer common channel lengths observed in older patients may represent dimensional changes over time rather than greater initial severity. Therefore, residual bias related to age at measurement cannot be completely excluded in our analysis.

As a single-center retrospective study with a small sample size, this research has inherent limitations. Follow-up durations varied among patients, and reliance on electronic medical records introduces potential bias due to incomplete or inconsistent data. Given the small cohort size, some statistically significant findings may have occurred by chance rather than reflecting true trends. Therefore, the primary value of this study may lie in its qualitative insights rather than its quantitative conclusions. In addition, loss to follow-up before the onset of puberty in a subset of patients limits the accurate assessment of menstruation outcomes, as some patients classified as non-menstruating may not have reached menarche during the observation period.

Furthermore, the indications guiding the timing of vaginoplasty, timing of anorectoplasty, and selection of surgical techniques were not standardized and may have varied over time, representing important limitations that could influence outcomes.

This study offers several valuable insights. Unlike previous research that categorized timing into early and late, we divided patients into three groups, allowing for a more nuanced comparison. This innovative approach evaluates outcomes based on both surgical timing and anatomical characteristics. While we recognize that this study cannot definitively recommend early or late vaginoplasty due to patient population heterogeneity and the multifaceted nature of surgical outcomes, it does provide important direction. Given the significant diversity of cloacal anomalies and the complexity of surgical decision-making, it is unlikely that a single optimal timing for vaginoplasty exists. Therefore, decisions will continue to depend on the clinical judgment of experienced surgeons. We hope this study will contribute to the evidence base that may eventually lead to more standardized guidelines.

Looking ahead, large-scale multicenter studies that include more cases and take into account anatomical complexity, surgeon experience, and patient or parental preferences are essential. While prospective studies may not be feasible for this rare and variable condition, robust retrospective multicenter research could clarify outcome trends and foster consensus on surgical timing.

In conclusion, determining the ideal timing for vaginoplasty in patients with cloacal anomalies remains a challenge. Our study suggests that patients who underwent delayed vaginoplasty after puberty may experience higher rates of vaginal complications. Although establishing temporal causality is limited by the retrospective design, our findings highlight the need for further large-scale studies to inform clinical decision-making and the development of standardized timing guidelines.

## Data Availability

No datasets were generated or analysed during the current study.
